# Ultrafast optical response and ablation mechanisms of molybdenum disulfide under intense femtosecond laser irradiation

**DOI:** 10.1038/s41377-020-0318-8

**Published:** 2020-05-06

**Authors:** Changji Pan, Lan Jiang, Jingya Sun, Qingsong Wang, Feifei Wang, Kai Wang, Yongfeng Lu, Yeliang Wang, Liangti Qu, Tianhong Cui

**Affiliations:** 1 Laser Micro/Nano-Fabrication Laboratory, School of Mechanical Engineering, Beijing Institute of Technology, 100081 Beijing, P.R. China; 2 Department of Electrical Engineering, University of Nebraska-Lincoln, Lincoln, NE 68588-0511 USA; 3 School of Information and Electronics, Beijing Institute of Technology, 100081 Beijing, P.R. China; 4 Key Laboratory for Advanced Materials Processing Technology, Ministry of Education of China, Department of Mechanical Engineering, Tsinghua University, 100084 Beijing, P.R. China; 5 Department of Mechanical Engineering, University of Minnesota, Minneapolis, MN 55455 USA

**Keywords:** Laser material processing, Ultrafast photonics

## Abstract

Numerous valuable studies on electron dynamics have focussed on the extraordinary properties of molybdenum disulfide (MoS_2_); however, most of them were confined to the level below the damage threshold. Here the electron dynamics of MoS_2_ under intense ultrafast laser irradiation was investigated by experiments and simulations. Two kinds of ablation mechanisms were revealed, which led to two distinct types of electron dynamics and final ablation morphology. At a higher fluence, the emergence of superheated liquid induced a dramatic change in the transient reflectivity and micro-honeycomb structures. At a lower fluence, the material was just removed by sublimation, and the ablation structure was relatively flat. X-ray photoelectron spectroscopic (XPS) measurements demonstrated that thermal decomposition only occurred at the higher fluence. Furthermore, a theoretical model was developed to deeply reveal the ultrafast dynamics of MoS_2_ ablation. The simulation results were in good agreement with the temporal and spatial reflectivity distribution obtained from the experiment. The electron and lattice temperature evolution was also obtained to prove the ablation mechanism. Our results revealed ultrafast dynamics of MoS_2_ above the damage threshold and are helpful for understanding the interaction mechanism between MoS_2_ and intense ultrafast lasers, as well as for MoS_2_ processing applications.

## Optoelectronics: Digging into molybdenum disulfide

Ultrafast damage-causing laser pulses affect bulk molybdenum disulfide differently depending on their energy per area, revealing further insight into the ultrafast electron dynamics. The research group of Lan Jiang from Beijing Institute of Technology, in cooperation with Tianhong Cui of University of Minnesota, has predicted and examined what happened to molybdenum disulfide on electron and lattice level when intense femtosecond laser pulses irradiate. Lower damage-causing energies per area, known as fluences, removed material through inducing a solid-to-gas transition. Higher fluences caused superheating, liquidation, and the formation of nanocracks and nanoridges. Tests on electron dynamics obtained by the materials’ reflectivity with different fluences were followed by theoretical simulations, which further helped to reveal the electron and lattice temperature evolution. The findings further understandings for laser processing of molybdenum disulfide and for its use in optoelectronics.

## Introduction

Molybdenum disulfide (MoS_2_) has attracted considerable research attention because of its potential applications in field effect transistors^1,2^, optoelectronic devices^3^, and electrocatalysts^4,5^, among others. The great potential of MoS_2_ is attributed to its novel and intriguing physical, chemical, and mechanical properties^6–9^, the most prominent of which include the emergence of a direct bandgap, strong light–matter interactions, and enhanced catalytic activity^10^. When MoS_2_ is applied in optoelectronics, the electron dynamics must be understood in detail because the ultrafast electron dynamics is known to determine the electronic transport and optical properties of semiconductors^11^. For instance, the intraband relaxation rate was revealed to increase by >40-fold in the monolayer compared to that observed in thick crystals^12^. Through time-resolved photoluminescence measurements, the carrier recombination time at low temperature was reported to be on a few picosecond time scale^13^, and insignificant polarization decay was observed over the entire emission duration^14^. In addition to its importance in optoelectronics applications, the ultrafast electron dynamics also plays a vital role in the laser fabrication of MoS_2_. To detect the ultrafast electron dynamics of MoS_2_, the ultrafast laser pump–probe technique is a useful tool. Numerous valuable studies on electron dynamics have already focussed on the attractive properties of MoS_2_^11,12,15–21^; however, they all studied free electron density below the level of 10^19^ cm^−3^. A study of the electron dynamics for a free electron density exceeding the damage threshold has not yet been proposed. At a laser fluence higher than the damage threshold, the electron dynamics may differ from that at a lower energy excitation. Therefore, it is necessary to provide deeper insights into the electron dynamics when an ultrafast intense laser pulse is applied.

In this study, the electron dynamics of bulk MoS_2_ under the irradiation of an intense ultrafast laser pulse was investigated through both experiments and simulations. The pump–probe technique was used to image the excited surface of MoS_2_. The transient reflectivity was recorded and analysed for both the time and space domains. Two distinct types of transient reflectivity were observed, which corresponded to two types of final ablation structures. X-ray photoelectron spectroscopic (XPS) analysis was carried out for the final structures. Based on these observations, two kinds of ablation mechanisms are proposed. Simultaneously, a model was developed for MoS_2_ to simulate the electron and lattice dynamics, as well as the reflectivity. The results obtained from the experiment and simulation were compared and proved to be in good agreement. By analysing the electron dynamics and final ablation structures, two kinds of structure formation mechanisms were revealed for MoS_2_ under intense ultrafast laser irradiation with distinct fluences.

## Results

### Ultrafast reflectivity dynamics analysis based on experimental data

**Fig. 1:**
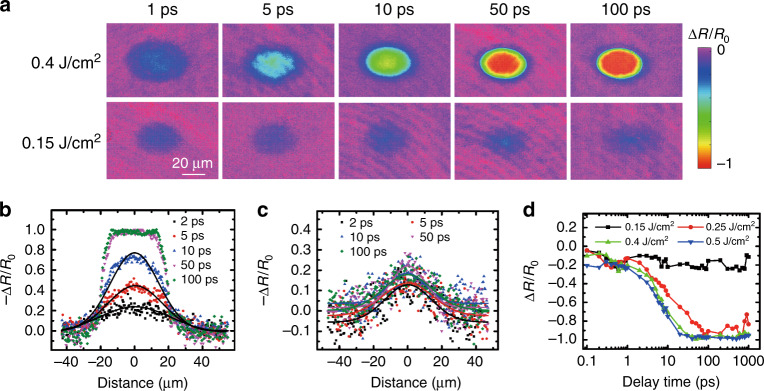
Ultrafast reflectivity dynamics after laser excitation with various fluences. **a** 2D mapping of the transient reflectivity at delay times from 1 to 100 ps under irradiation with fluences of 0.4 and 0.15 J/cm^2^. **b** Spatial reflectivity distribution extracted from the long axis of the focal area at the 0.4 J/cm^2^ fluence in **a**. **c** Spatial reflectivity distribution extracted from the long axis of the focal area at the 0.15 J/cm^2^ fluence in **a**. The solid lines in **b** and **c** are the Gaussian-fitted lines based on the experimental data points. **d** Reflectivity dynamics at the focal spot centre for various laser fluences.

By using the set-up described in the “Materials and methods” section, the transient reflectivity was recorded at various delay times. The measured reflectivity was extracted in the form of the normalized reflectivity change,$\Delta R/R_0 = \left( {R - R_0} \right)/R_0$, where *R* and *R*_0_ can be represented by the intensities of a shadowgraph image with and without laser irradiation, respectively. Figure 1 presents the ultrafast reflectivity dynamics under irradiation of a femtosecond laser with various fluences. In Fig. 1a, two-dimensional (2D) transient reflectivity images are presented to describe the electron dynamics after femtosecond laser excitation. The reflectivity dynamics after laser excitation is extremely dissimilar for the fluences of 0.4 and 0.15 J/cm^2^. For the higher fluence, the normalized reflectivity change dramatically decreases as the delay time increases. The red area, which emerged after 50 ps, indicates a strong absorption process for the probe pulse. This strong absorption process was attributed to the superheating of liquid (named the overshooting phenomenon) in previous works^22–24^. By contrast, the normalized reflectivity change only gently decreases for the lower fluence (0.15 J/cm^2^) and remains relatively unchanged within a 100-ps delay time. The dramatic difference between these two fluences indicates two types of electron dynamics during femtosecond laser irradiation.

**Fig. 2:**
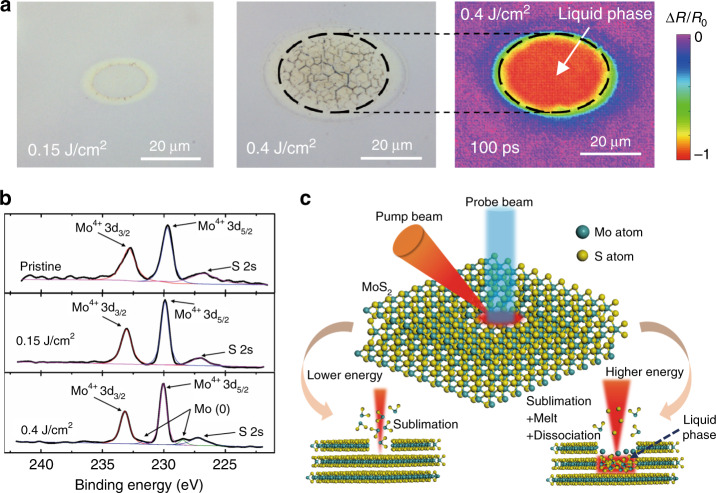
Ablation mechanisms of MoS_2_ under an intense femtosecond laser pulse. **a** Optical images of the final structures and comparison with the transient reflectivity image at a 100 ps delay time. **b** Comparison of the XPS Mo 3d spectra of pristine and laser-treated MoS_2_. **c** Schematic of ablation processes in different fluence cases.

For a detailed discussion, as displayed in Fig. 1b, c, the reflectivity distribution along the long axis of the elliptical focal area was extracted from each related delay time image presented in Fig. 1a for the 0.4 and 0.15 J/cm^2^ fluences. At the higher fluence, the reflectivity presents a Gaussian distribution before a 10-ps delay time; however, a flat-top profile emerges after a 50-ps delay time. The width of the flat-top profile does not change when the delay time increases to 100 ps, which means that there is a critical fluence that causes absorption saturation. In the lower fluence case, as illustrated in Fig. 1c, the reflectivity distribution can also be fitted by a Gaussian distribution even up to a 100-ps delay time, although the signal-to-noise ratio is not very high. No flat-top profile emerges in the lower fluence case, which implies no absorption saturation. To further reveal the reflectivity dynamics, a series of fluences were applied to excite the sample, and the reflectivity dynamics of the focal centre are plotted in Fig. 1d. The normalized reflectivity change evolution is consistent with the phenomenon observed in Fig. 1a. It is evident that the reflectivity dynamics can be easily classified into two types of mechanisms. In the case of the lower fluence, the normalized reflectivity change fluctuates around the value of −0.2. However, at the higher fluence, the normalized reflectivity change is reduced to nearly −1, which means that the probe beam has been almost completely absorbed. It is necessary to emphasize that both fluences are above the damage threshold, which can be verified by the final morphology image in Fig. 2. Inspired by these two distinct types of reflectivity dynamics, it is reasonable to speculate that distinct electron dynamics lead to dissimilar ablation structures. To verify this assumption, the final structures after laser ablation were investigated by using optical microscopy (OM) and atomic force microscopy (AFM).

### Ablation mechanism of MoS_2_ under ultrafast laser irradiation

Under irradiation by an intense laser pulse, material removal is of course a matter. However, the final structures induced by material removal can be relatively diverse and are associated with the electron and lattice dynamics^25^. Considering the marked difference in the reflectivity and electron dynamics between the lower and higher fluences, the structures after laser excitation should differ between these two cases. In Fig. 2a, the optical images of the final structures after laser ablation are compared for the two typical fluences. For both fluences, the region of material removal presents an elliptical shape due to the oblique incidence of the pump pulse. A pale yellow ellipse-shaped ring region surrounds the focal area, which might arise from laser-induced material modification. However, the structures are relatively dissimilar in the central area for the two fluences. For the higher fluence, micro-honeycomb structures are observed within a smaller ellipse nested within the yellow ring. For the lower fluence, there are no micro-honeycomb structures within the central ellipse region. The colour of the central ellipse is the same as that of the original region, which indicates that the remaining structure is still flat after ablation. To further study these structures, measurements were taken with AFM, and the results are presented in Fig. S1. As revealed by the AFM images, the micro-honeycomb structures observed in optical images (higher fluence in Fig. 2a) are composed of many nanoridges and nanocracks. Most nanoridges and nanocracks cross each other with an angle of 120°. To reveal the relationship of the final structures with the electron and lattice dynamics, the optical image of the final structure is compared with the transient reflectivity image, shown in the rightmost image of Fig. 2a. The transient reflectivity image is selected from Fig. 1a at the 100 ps delay time. The dashed ellipse in the transient reflectivity image represents the reflectivity flat-top area, which indicates a strong absorption process. The dashed ellipse in the optical image represents the micro-honeycomb structure area. It is found that these two dashed ellipses are the same size. This perfect consistency indicates that the formation of micro-honeycomb structures may be associated with a strong absorption process.

Figure 2b presents a comparison of the XPS Mo 3d spectra of pristine and laser-treated MoS_2_. For all cases, a pair of binding peaks at Mo^4+^ 3d_3/2_ (233.18 eV) and Mo^4+^ 3d_5/2_ (230.5 eV) are obviously found, which originates from Mo^4+^ of MoS_2_ molecules. After the lower fluence (0.15 J/cm^2^) treatment, the XPS Mo 3d spectrum is nearly unchanged compared to that of pristine MoS_2_. However, another pair of binding peaks at 228.46 and 231.59 eV emerges for the higher fluence (0.4 J/cm^2^) treatment. These binding peaks can be assigned to Mo 3d_3/2_ and Mo 3d_5/2_ of Mo metal^26^, which is the chemical reaction product of MoS_2_ decomposition. The thermal decomposition temperature of MoS_2_ is 1873 K, at which temperature decomposition occurs with the sulfur evolving as gas and the molybdenum remaining^27^.

$${\mathrm{MoS}}_2\mathop{\longrightarrow}\limits^{{1873\,{\mathrm{K}}}}{\mathrm{Mo}} + {\mathrm{S}}\left( \uparrow \right)$$

According to the XPS results, thermal decomposition only occurs in the higher fluence case. For the higher fluence, the lattice temperature of MoS_2_ can be heated to above the decomposition temperature of MoS_2_. In contrast, the heated lattice temperature for the lower fluence is just below the decomposition level. Thus the XPS spectrum of Mo metal cannot be observed for the lower fluence.

By analysing these results, two kinds of ablation mechanisms are proposed when an intense laser pulse is applied to excite MoS_2_, where the difference is attributed to the emergence of superheating of the liquid phase. A schematic of the ablation mechanisms for the different fluence cases is given in Fig. 2c. In the higher fluence case, the lattice temperature can be heated to a higher temperature. When the lattice is heated to above the melting point of MoS_2_, the bulk MoS_2_ will melt to the liquid phase. Owing to the emergence of the liquid phase, the transient reflectivity dramatically changes, which causes a strong absorption process, as described in Fig. 1a. Furthermore, MoS_2_ dissociation also occurs at such high temperature, where the sulfur evolves as gas and the molybdenum remains. After a temperature balance is achieved between the electrons and lattice, a high-temperature liquid phase layer is formed and releases energy to the surrounding solid phase. During the resolidification process, thermal stress is easily caused by the temperature inhomogeneity, which leads to the formation of ridges and cracks^28,29^. The specific intersection angle (120°) of the ridges and cracks might be associated with the lattice structure of MoS_2_. By contrast, for the lower fluence, the lattice cannot be heated to above the melting point of MoS_2_. Therefore, neither melting nor resolidification occurs in the lower fluence case, where the bulk MoS_2_ is removed by sublimation. In this case, the bottom is smoother for the lower fluence than that for the higher fluence. The detailed structures were imaged by scanning electron microscopy, as shown in Fig. S2, which provides more information on the phase transition difference between these two fluences. This analysis can be further confirmed through theoretical calculations of the lattice temperature evolution.

### Theoretical model

**Fig. 3:**
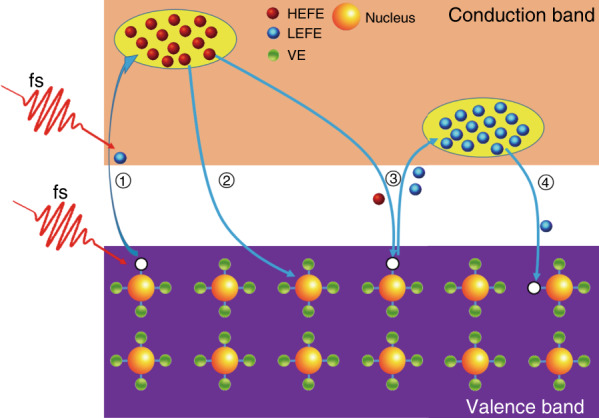
Scheme of electron and lattice out-of-equilibrium dynamics interactions. ① Laser-induced ionization and free electron heating; ② high-energy electron-induced lattice heating; ③ high-energy electron-induced ionization; ④ free electron recombination. HEFE high-energy free electron, LEFE low-energy free electron, VE valence electron.

To provide deeper insights into the mechanism of MoS_2_ ablation, a theoretical model is built to simulate the electron and lattice dynamics during and after intense femtosecond laser irradiation. Because of the ultrashort duration of the femtosecond laser pulse, the laser–material interaction process occurs over an ultrashort time scale and induces transient out-of-equilibrium plasma. As schematically represented in Fig. 3, we mainly consider four interactions over several hundred picoseconds as follows: (1) laser-induced ionization and free electron heating, (2) high-energy electron-induced lattice heating, (3) high-energy electron-induced ionization, and (4) free electron recombination. The extremely intense electromagnetic field of the femtosecond laser can generate a mass of free electrons from the valence band to the conduction band via ionization. Accompanying ionization, the ionized electrons are heated to a high temperature^30–32^, thus storing most of the energy of the laser pulse. During laser pulse irradiation, the lattice does not have sufficient time to effectively respond to the electromagnetic field and remains cool. After laser irradiation, the hot electrons interact with the cool lattice and valence electrons. Finally, the system relaxes towards temperature equilibrium within tens of picoseconds.

First, we consider modelling the process of laser-induced ionization and free electron heating. Because the bandgap of bulk MoS_2_, 1.2 eV, is lower than the photon energy, 1.5 eV, one-photon ionization is considered the main method of generating free electrons. The following single rate equation is applied to calculate the free electron generation:^33,34^

1 $$\frac{{\partial n_{\rm{f}}}}{{\partial t}} = P\left( I \right) - \frac{{n_{\rm{f}}}}{{\tau _{{\rm{re}}}}}$$

where *n*_f_ is the free electron density, *t* is the time, *τ*_re_ is the free electron recombination time, *P*(*I*) is the photoionization term, and *I* is the laser intensity. The free electron recombination time *τ*_re_ is considered a constant value of 180 ps, as measured for MoS_2_ in a previous work^18^. The photoionization term can be calculated by the perturbation formula for one-photon absorption^35,36^. Meanwhile, the ionized free electrons are also heated by the intense electromagnetic field during laser irradiation. The electron temperature during laser irradiation can be calculated using the following expression^34^:

2 $$c_{\rm{e}}n_{\rm{f}}\frac{{\partial T_{\rm{e}}}}{{\partial t}} = {\it{\upalpha }}_{\rm{h}}I$$

where *c*_e_ is the specific heat of free electrons, *T*_e_ is the free electron temperature, and *α*_h_ is the free electron absorption coefficient, which can be deduced from the free electron dielectric function. The specific heat of the free electrons can be deduced from the Fermi distribution^32^. Based on Eqs. (1) and (2), we can obtain the free electron density and temperature after laser pulse excitation. In this case, the energy of the laser pulse is transferred to the high-energy free electron system. Subsequently, the high-energy free electrons transfer energy to lattice and valence electrons through high-energy electron-induced lattice heating and valence electron ionization.

To describe lattice heating, the two-temperature model (TTM) is used to calculate the energy transfer between high-energy electrons and the lattice as follows^32,37^:

3 $$c_{\rm{e}}\frac{{\partial T_{\rm{e}}}}{{\partial t}} = {\it{\nabla }}\left[ {{\it{\upkappa }}_{\rm{e}}{\it{\nabla }}T_{\rm{e}}} \right] - G\left( {T_{\rm{e}} - T_{\rm{l}}} \right) + S$$

4 $$c_{\rm{l}}\frac{{\partial T_{\rm{l}}}}{{\partial t}} = G\left( {T_{\rm{e}} - T_{\rm{l}}} \right)$$

where *κ*_e_ is the free electron heat conductivity, *G* is the electron–lattice coupling factor, *T*_l_ is the temperature of the lattice, *S* is the laser source term, and *c*_l_ is the specific heat of the lattice. Because the processes of lattice heating and laser excitation are separated in the time domain, the laser source term, *S*, in Eq. (3) is ignored in the simulation. The free electron heat conductivity can be obtained as 4.2 cm^2^/s from experimental measurements^18^.

By modelling these processes, the electron and lattice dynamics can be simulated, including the electron density, electron temperature, lattice temperature, and transient reflectivity. More detailed information on the model is given in Supplementary Information.

### Comparison of simulation with experiment

**Fig. 4:**
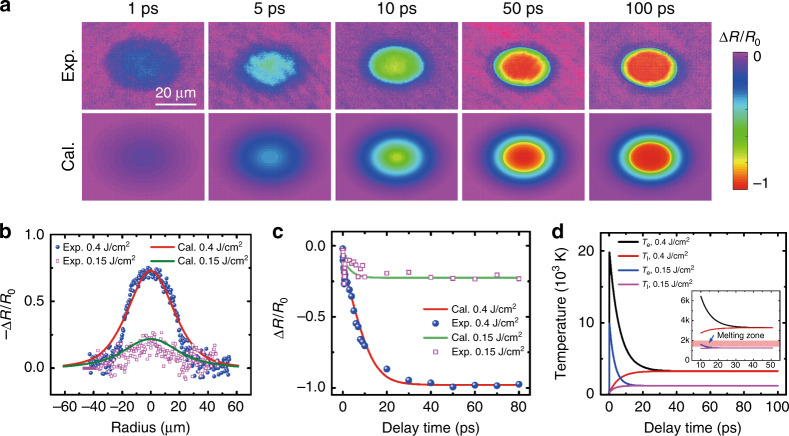
Analysis of simulation and experimental results. **a** Comparison of 2D transient reflectivity mapping between simulation and experimental results. **b** Spatial distribution of transient reflectivity extracted from the long axis of the focal spot at a 10-ps delay time for both the experiment and calculation. **c** Temporal evolution of transient reflectivity extracted from the focal centre for both the experiment and calculation. **d** Electron temperature and lattice temperature evolution after excitation with different fluences (0.4 and 0.15 J/cm^2^). The inset presents the details of the curve evolution from 10 to 50 ps.

By applying the model described above, the electron and lattice dynamics after intense laser pulse irradiation can be simulated. Based on the electron and lattice dynamics, the transient optical properties can be obtained, including the ultrafast reflectivity evolution. To verify the feasibility of the model, the calculated transient reflectivity images are plotted for comparison with the measured images for the higher laser fluence case. As Fig. 4a illustrates, the 2D transient reflectivity images obtained through calculation are all well consistent with those obtained through measurement from 1 to 100 ps. The absorption saturation caused by the superheating phenomenon is observed for both the simulation and measurement results.

Figure 4b presents the calculated spatial distribution of the normalized reflectivity change, which was plotted for comparison with the experimental results. The calculated and measured normalized reflectivity change distributions were extracted at 10 ps for two typical laser fluences (i.e., 0.4 and 0.15 J/cm^2^). The reflectivity points along the long axis of the elliptical focal area were extracted. The distribution profiles at these two fluences both present a Gaussian-like distribution, which arises from the Gaussian distribution of the laser pulse intensity. Although background noise exists in the measurements, which is particularly evident for the lower fluence, the curves calculated through the model are in good agreement with the data points obtained through the measurements.

In addition to the spatial distribution of the transient reflectivity, the normalized reflectivity change evolution at the spot centre was also extracted both for the calculation and measurement results at the two laser fluences. As Fig. 4c illustrates, the calculation results are also in good agreement with the measurement results. In both the calculation and measurement results, the normalized reflectivity change decreases with increasing delay time after laser excitation, different from the results of other semiconductors, such as Ge^23^ and Si^38^. This difference may be attributed to the competition between the original valence electron and excited free electron contributions. When the intense laser is focussed onto the materials, numerous valence electrons are ionized to the conduction band as free electrons. With the free electron increase, the population of valence electrons rapidly decreases, and their contributions to the dielectric function should be reconsidered. The free electron density evolution was calculated for both laser fluences, as shown in Fig. S3. It is worth noting that the normalized change evolves to a stabilized state faster for the lower fluence than for the higher fluence. The reflectivity at the lower fluence is reduced to approximately −0.25 after 10 ps and maintained at this value up to 80 ps or even longer, as shown in Fig. 1d. By contrast, it takes approximately 25 ps to realize reflectivity stabilization under the higher fluence, and the reflectivity decreases down to nearly −1.0. The time for reflectivity stabilization should be associated with the electron dynamics relaxation processes, that is, the electron and lattice temperature balance.

The lattice temperature is important in laser ablation and can affect the phase change. For the early time within a picosecond after the laser pulse arrives, the lattice is believed to remain cool^39^. When the excitation time reaches the picosecond scale, the energy transfer from the electron system to the lattice system should be considered because of electron–phonon scattering^24,32^. This process can be well described by the TTM. Figure 4d illustrates the calculated evolution of the electron temperature and lattice temperature after pulse irradiation with two typical fluences. After laser pulse irradiation, the electrons are heated to a high temperature level for both fluences. In contrast, the lattice remains cool at room temperature. Subsequently, the energy transfer from the free electrons to the lattice is initiated through electron-phonon scattering because of the large temperature difference. During this process, the electron temperature rapidly decreases, and the lattice is strongly heated. It is worth noting that the electron temperature and lattice temperature achieve a balance after 25 ps for the higher fluence and 10 ps for the lower fluence. These two characteristic times for temperature relaxation are consistent with the times of reflectivity relaxation, which indicates that the transient reflectivity is mainly affected by the electron temperature and lattice temperature. The inset in Fig. 4d presents the details of the temperature evolution from 10 to 50 ps. The light red rectangle in the inset represents the melting point zone of MoS_2_ (1458–2073 K) according to previous works^40,41^. Evidently, for the higher laser fluence, the lattice can be heated to a much higher temperature than the melting point of MoS_2_, which confirmed the analysis regarding the superheating phenomenon^23^. However, for the lower fluence, the lattice temperature is just below the melting point of MoS_2_. Therefore, as presented in Fig. 1a, no strong absorption is caused by superheating after laser excitation at the lower fluence. Even though the lattice temperature is slightly below the melting point in the lower fluence case, material removal still occurs through sublimation because bulk MoS_2_ can sublimate when the temperature exceeds 698 K^42^. In addition, the much higher lattice temperature also causes thermal decomposition, which is well consistent with the XPS results in Fig. 2c.

## Discussion

In summary, through experiments and theory, the electron dynamics of bulk MoS_2_ under irradiation with an intense ultrafast laser was investigated. In the experiment, the pump–probe technique was used to detect the excited surface of bulk MoS_2_. Using this technique, the transient reflectivity was recorded and analysed for both the time and space domains under irradiation with various laser fluences. The reflectivities were found to be quite different, where strong absorption emerged for the higher fluence. After real-time detection, the final morphology after laser pulse excitation was also examined by OM and AFM. For the higher fluence, micro-honeycomb structures composed of nanoridges and nanocracks were observed in the central area. However, the central area ablated by the lower fluence was relatively flat. Furthermore, XPS analysis was carried out, which indicated that thermal decomposition only occurred in the higher fluence case. By analysing these results, two kinds of ablation mechanisms were proposed, where the dramatic difference between the two fluences was attributed to the superheating phenomenon. A theoretical model was developed for bulk MoS_2_ to simulate the electron and lattice dynamics, as well as the reflectivity. The reflectivity dynamics obtained from the simulation was in good agreement with the experimental results. In addition, the electron and lattice temperature dynamics was calculated to confirm the emergence of the superheating phenomenon. The results are helpful for understanding the mechanism of the interaction between MoS_2_ and intense ultrafast laser, which might especially promote the potential applications of intense ultrafast laser on MoS_2_.

## Materials and methods

### Sample preparation

The MoS_2_ samples used for this study were prepared by mechanical exfoliation from naturally occurring crystals (purchased from Nanjing MKNANO Tech. Co., Ltd). The thickness of every sample was >10 μm.

### AFM measurement

The surface morphology after laser excitation was measured by AFM (Bruker Dimension Edge).

### Scanning electron microscopy measurement

The surface morphology after laser excitation was measured by scanning electron microscopy (COXEM EM-30N).

### XPS measurement

XPS was carried out using a PHI Quantera X-ray photoelectron spectrometer.

### Ultrafast dynamics detection

To detect the time-resolved reflectivity of bulk MoS_2_, a pump–probe technique was used to image the transient surface of samples under intense femtosecond laser irradiation. The scheme of the experimental set-up is displayed in Fig. S4. Pulses generated from a Ti:sapphire chirped pulse amplification system (Spectra-Physics Spitfire Ace) with a wavelength, *λ*, of 800 nm and a pulse duration of 35 fs were split into pump and probe pulses by a beam splitter. The pump pulse was focussed onto the sample surface with a lens (L1, focal length of 150 mm) at a 45° angle of incidence. The probe pulse was guided to pass through an optical delay line, and its frequency was doubled to 400 nm using a beta barium borate crystal. The residual 800 nm radiation was filtered by a bandpass filter. The probe pulse was then focussed by a lens (L2, focal length of 150 mm) to the back focal plane of a long-working-distance microscope objective (MO: ×10, NA = 0.4). Before entering the MO, the probe pulse passed through a beam splitter. The MO finally collimated this pulse such that it could be used for normal incidence illumination of the sample surface. After illuminating the sample, the reflected fraction of the probe pulse passed through the MO again and was reflected by the beam splitter in front of the MO. Then a tube lens was used to focus the reflected probe pulse onto a charge-coupled device camera to image the transiently excited surface. To determine the reflectivity change during laser irradiation, two shadowgraph images should be taken at every position of the sample. One of them should be taken when the pump pulse is blocked, which is set as the background reflectivity image, *R*_0_. The other should be taken when the sample is exposed to the pump pulse at a delay time, which is treated as the laser-excited reflectivity image, *R*. Based on the two shadowgraph images, we can calculate the normalized reflectivity change $\Delta R/R_0 = \left( {R - R_0} \right)/R_0$. In this case, the temporal and spatial evolution of the laser-induced surface reflectivity can be detected with high precision.

## Supplementary information

Supplementary information
