# Supplementary material for: Ultrafast optical response and ablation mechanisms of molybdenum disulfide under intense femtosecond laser irradiation
Source: Light Sci Appl. 2020 May 6;9:80. doi: 10.1038/s41377-020-0318-8 (PMC7203173; doi:10.1038/s41377-020-0318-8)
Supplement: Supplementary file 1 — Supplementary information [file 41377_2020_318_MOESM1_ESM.docx]

**Supplementary Information for**

**Ultrafast Optical Response and Ablation Mechanisms of Molybdenum Disulfide under Intense Femtosecond Laser Irradiation**

Changji Pan^1^, Lan Jiang^1^*, Jingya Sun^1^, Qingsong Wang^1^, Feifei Wang^1^, Kai Wang^1^, Yongfeng Lu^2^, Yeliang Wang^3^, Liangti Qu^4^ and Tian-Hong Cui^5^

^1^Laser Micro/Nano-Fabrication Laboratory, School of Mechanical Engineering, Beijing Institute of Technology, Beijing 100081, PR China.

^2^Department of Electrical Engineering, University of Nebraska-Lincoln, Lincoln, NE 68588-0511, USA

^3^School of Information and Electronics, Beijing Institute of Technology, Beijing 100081, China

^4^Key Laboratory for Advanced Materials Processing Technology, Ministry of Education of China, Department of Mechanical Engineering, Tsinghua University, Beijing 100084, P.R. China

^5^Department of Mechanical Engineering, University of Minnesota, Minneapolis, MN 55455, USA

Corresponding Email: [jianglan@bit.edu.cn](mailto:jianglan@bit.edu.cn)

1. **AFM and SEM images of final structures**

**
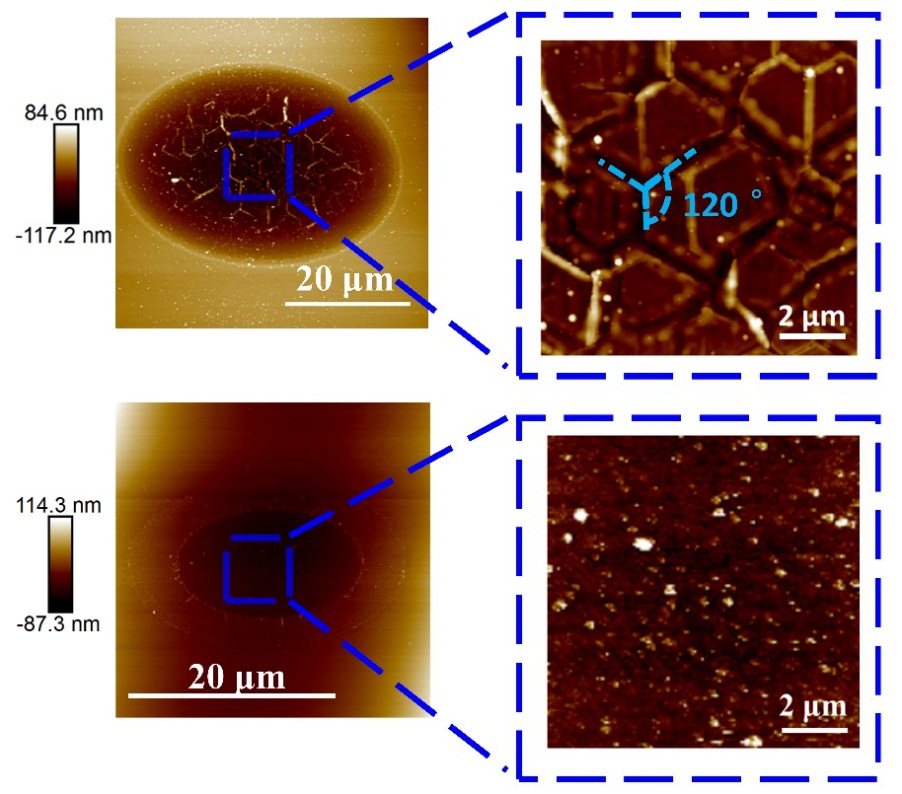
**

**Fig. S1** AFM images of final ablation structures. Two inserts present more detail information for the center area. The blue dash line in upper insert indicates the nanoridges and nanocracks cross each other with an angle of 120°.


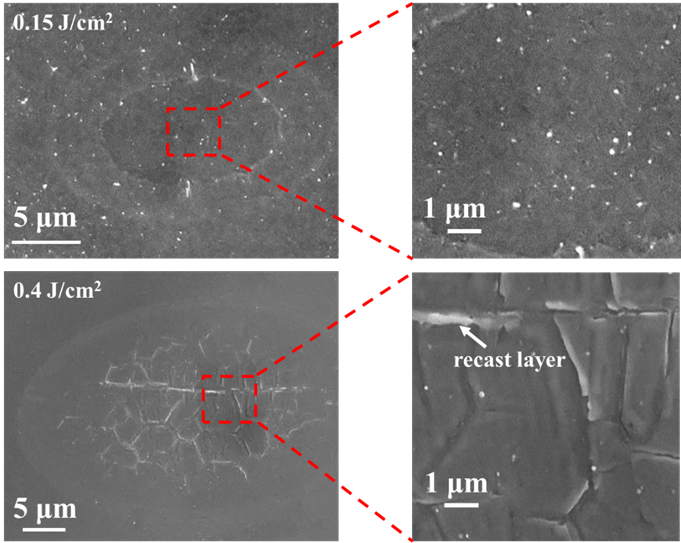


**Fig. S2** SEM images of ablation structures at 0.15 Jcm^-2^ and 0.4 Jcm^-2^.

1. **Electron density evolution**

**
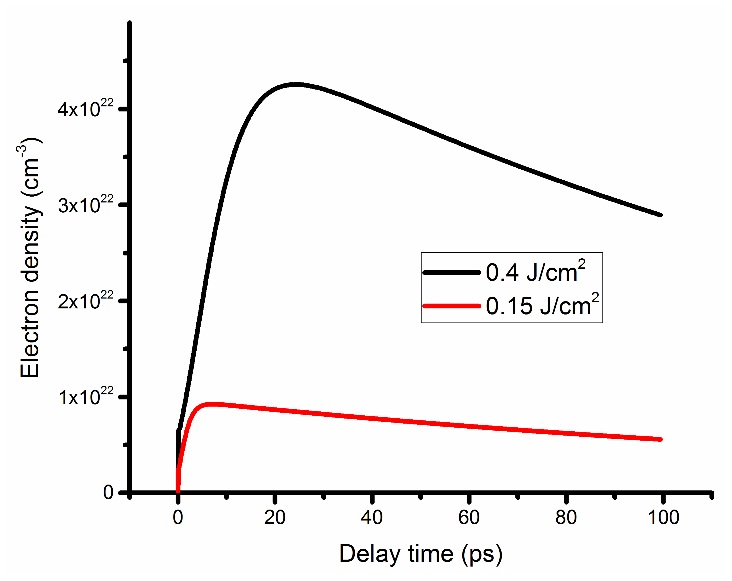
**

**Fig. S3** The free electron density evolution induced by two typical fluence (0.4 and 0.15 Jcm^-2^).

1. **Pump probe setup**

**
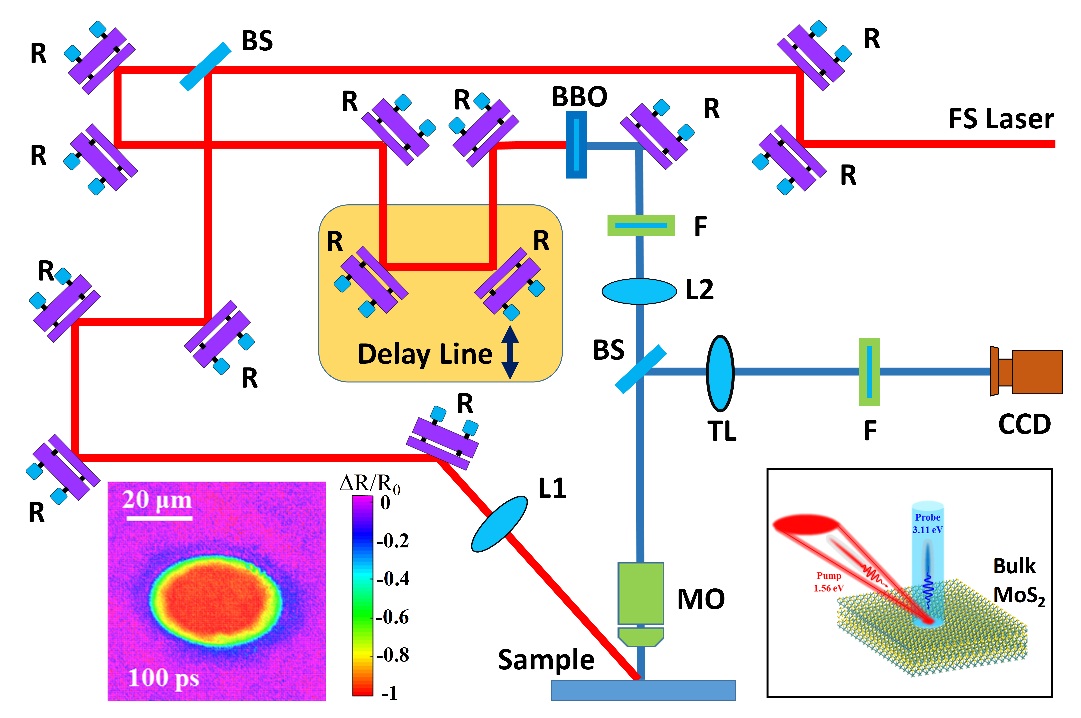
**

**Fig. S4** Scheme of the experimental setup for the measurement of time-resolved reflectivity. R, reflector; BS, beam splitter; L1, lens (focus length 150 mm); L2, lens (focus length 150 mm); BBO, beta barium borate crystal; F, bandpass filter; MO, long-working-distance microscope objective; CCD, charge-coupled device camera; The right inset shows a simplified scheme of the regions interacting with the pump and probe pulses and the left inset is a typical image of reflectivity mapping in the excited region at 100 ps delay time.

1. **Detail information of model**

First, we should model laser induced ionization and free electron heating. Because the bandgap of bulk MoS_2_, 1.2 eV, is lower than the photon energy, 1.5 eV, one-photon ionization is considered as the main method to generate free electrons. The following single rate equation is applied to calculate the free electron generation: ^1-2^

$\frac{\partial n_{f}}{\partial t}=P\left( I \right)-\frac{n_{f}}{\tau_{re}}$ (1)

where $n_{f}$ is the free electron density, *t* is the time,$\tau_{\mathrm{re}}$ is the free electron recombination time, $P\left( I \right)$ is the photoionization term, and *I* is the laser intensity. The free electron recombination time $\tau_{\mathrm{re}}$ is considered a constant value at 180 ps, as measured for MoS_2_ in a previous work.^3^ The photoionization term can be calculated by perturbation formula for one-photon absorption^4-5^ as follows:

$P= \frac{e^{2}N_{ph}\sqrt{m^{*}}\sqrt{E_{g}}}{\hbar^{2}\varepsilon_{\infty}}\sqrt{{(\frac{\hbar\omega}{E_{g}})}^{2}-1}$ (2)

where $e$ is the electron charge, $N_{\mathrm{ph}}$ is the number of incident photons per unit volume, $m^{*}$ is the reduced effective mass of the electron, $E_{g}$ is the bandgap of MoS_2_, $\hbar$ is the reduced Planck constant, $\varepsilon_{\infty}$ is the original dielectric constant, and $\omega$ is the laser frequency. Meanwhile, the ionized free electrons are also heated by an intense electromagnetic field during laser irradiation. The electron temperature during laser irradiation can be calculated using the following expression:^2^

$c_{e}n_{f}\frac{\partial T_{e}}{\partial t}=\alpha_{h}I$ (3)

where $c_{e}$ is the specific heat of free electrons, $T_{e}$ is the free electron temperature, and $\alpha_{h}$ is the free electron absorption coefficient, which can be deduced from the free electron dielectric function. The specific heat of the free electrons can be deduced from the Fermi distribution.^6^

Based on eqs. 1–3, we can obtain the values of free electron density and temperature after laser pulse excitation. In this case, the energy of the laser pulse is transferred to the high energy free electron system. Subsequently, the high energy free electrons transfer energy to lattice and valence electrons through high energy electron induced lattice heating and valence electron ionization.

To describe lattice heating, the famous two-temperature model (TTM) was used to calculate the energy transfer between high energy electrons and lattice as follows: ^6-7^

$c_{e}\frac{\partial T_{e}}{\partial t}= \nabla\left[ \kappa_{e}\nabla T_{e} \right]-G\left( T_{e}-T_{l} \right)+S$ (4)

$c_{l}\frac{\partial T_{l}}{\partial t}=G(T_{e}-T_{l})$ (5)

where $\kappa_{e}$ is the free electron heat conductivity, $G$ is the electron-lattice coupling factor, $T_{l}$ is the temperature of the lattice, $S$ is the laser source term, and $c_{l}$ is the specific heat of the lattice. Because the processes of lattice heating and laser excitation were separated in time domains, the laser source term, $S$, was ignored in the simulation presented in eq. 4. The free electron heat conductivity can be obtained as 4.2 cm^2^/s from experimental measurements.^3^ The electron-lattice coupling factor can be estimated using the following:^6, 8^

$G= \frac{\pi^{2}m_{e}n_{f}{c_{s}}^{2}}{6\tau_{el}T_{e}}$ (6)

where $m_{e}$ is the mass of the electron, and $c_{s}$ is the sound speed in bulk material. The sound speed can be obtained using $c_{s}=\sqrt{B/\rho}$, where $B$ is the bulk modulus, and $\rho$ is the mass density of bulk MoS_2_. The specific heat of the lattice can be deduced from the Debye model.^9-10^ The TTM can well simulate the process of energy transfer from high free electrons to lattice in picosecond time scale, which will further lead to melting and material remove.

Once electrons are excited into the conduction band, they will be heated by the intense electromagnetic field and obtain sufficient energy to excite other valence electrons.^1^ This process is called impact ionization or collisional ionization.^1-2^ Laser induced impact ionization has been investigated for many years,^1, 11-12^ which is considered to be driven by laser field. In these previous models, scientists only considered impact ionization during laser pulse irradiation. However, the free electrons still remain at a high temperature in the conduction band after femtosecond laser excitation, which also possess sufficient energy to enable the excitation of valence electrons. High energy electron induced ionization should be taken into account for its energy relaxing effect after femtosecond laser irradiation. The increasing free electron density and decreasing valence electron density both considerably influence the transient optical property, which is verified by our pump-probe experiment.

In a previous work on the laser induced superheat of a semiconductor, the plasma frequency $\omega_{p}$ was reported to increase with lattice temperature as follows:^13^

${\omega_{p}}^{-1}\frac{\partial\omega_{p}}{\partial T_{l}}=K_{c}$ (7)

where $K_{c}$ is a constant parameter, and the plasma frequency $\omega_{p}$ is a function of the free electron density $n_{f}$:^1, 13^

${\omega_{p}}^{2}=\frac{n_{f}e^{2}}{m_{e}\varepsilon_{0}}$ (8)

where $\varepsilon_{0}$ is the vacuum dielectric constant. Based on eqs. 7–8, we can obtain the relationship between free electron density and lattice temperature. Therefore, the electron density evolution can be calculated after laser pulse excitation.

When an electron is excited into the conduction band, it has the possibility to recombine to the valence band. Statistically, an excited free electron’s lifetime is dependent on various materials. For MoS_2_, the free electron lifetime was experimentally measured as 180 ps in a previous work.^3^ The lifetime is treated as a constant in our model so that the electron density can be calculated at any time, which will be used to estimate the transient optical property of MoS_2_.

The transient optical property can be described as the dielectric function of laser induced plasma. There are mainly two contributions to the dielectric function for semiconductors (*e.g.*, free electrons and valence electrons). In some previous works,^14-15^ the dielectric function change was only determined by the effect of free electrons, where the contribution of free electrons to the dielectric function is widely described by the Drude-Lorentz model.^1^ For transparent material, the contribution of valence electrons is so small that it can be ignored. However, the effect of valence electrons on the dielectric function should be taken into account, especially when valence electron density considerably decreases because of strong ionization. Therefore, to determine the contribution of valence electrons, the dielectric function should be expressed as follows:^16^

$\varepsilon= \left( 1+\frac{3(n_{0}-n_{f})\chi}{3-(n_{0}-n_{f})\chi} \right)-\frac{{\omega_{p}}^{2}}{\omega^{2}+i\omega\Gamma}$ (9)

where $\varepsilon$ is the dielectric function, $n_{0}$ is the original valence electron density in bulk MoS_2_, $\omega$ is the laser frequency, $\chi$ is the electric susceptibility derived from the Clausius–Mossotti relation,^17^ and $\Gamma$ is the free electron scattering rate.^1^ The free electron scattering rate is attributed to the effect of electron-lattice scattering and electron-electron scattering.^2^ When the dielectric function is determined, the time-resolved reflection can be calculated in the laser irradiated area as follows:^18^

$R=\frac{\left( n-1 \right)^{2}+\kappa^{2}}{\left( n+1 \right)^{2}+\kappa^{2}}$ (10)

where $n$ and $\kappa$ are the refraction index and the extinction coefficient, respectively, which can be deduced from the dielectric function by $n+i\kappa= \sqrt{\varepsilon}$.

Based on the discussion aforementioned, we combined single rate equation and two temperature model to account for the electron dynamics of bulk MoS_2_ during intense femtosecond laser pulse irradiation. This model can simulate the relaxation of laser induced plasma within hundreds of picoseconds. The simulation results were analyzed and compared with the experimental results. The related parameters used in the model were listed in Table S1.

Table S1. Parameters used in model

| $K_{c}$ | $\chi$ | $\varepsilon_{0}$ | $m^{*}$ | $B$ | $\rho$ |
| --- | --- | --- | --- | --- | --- |
| 8.2e-4 | 0.9672+0.1114i | 3.915+3.166i | 0.018 | 2.4e12 Nm^-2^ | 4.8 gcm^-3^ |

**References**

(1) Balling, P.; Schou, J., Femtosecond-Laser Ablation Dynamics of Dielectrics: Basics and Applications for Thin Films. Rep. Prog. Phys. **2013**, 76, 036502.

(2) Jiang, L.; Tsai, H. L., Energy Transport and Material Removal in Wide Bandgap Materials by a Femtosecond Laser Pulse. Int. J. Heat. Mass. Tran. **2005**, 48, 487-499.

(3) Kumar, N.; He, J.; He, D.; Wang, Y.; Zhao, H., Charge Carrier Dynamics in Bulk MoS_2_ Crystal Studied by Transient Absorption Microscopy. J. Appl. Phys. **2013**, 113, 133702.

(4) Vaidyanathan, A.; Walker, T. W.; Guenther, A. H.; Mitra, S. S.; Narducci, L. M., Comparison of Keldysh and Perturbation Formulas for One-Photon Absorption. Phys. Rev. B **1979**, 20, 3526-3527.

(5) Vaidyanathan, A.; Mitra, S. S.; Narducci, L. M.; Shatas, R. A., One-Photon Absorption in Direct Gap Semiconductors. Solid State Commun. **1977**, 21, 405-407.

(6) Jiang, L.; Tsai, H.-L., A Plasma Model Combined with an Improved Two-Temperature Equation for Ultrafast Laser Ablation of Dielectrics. J. Appl. Phys. 2008, 104, 093101.

(7) Cheng, C. W.; Wang, S. Y.; Chang, K. P.; Chen, J. K., Femtosecond Laser Ablation of Copper at High Laser Fluence: Modeling and Experimental Comparison. Appl. Surf. Sci. **2016**, 361, 41-48.

(8) Shin, T.; Teitelbaum, S. W.; Wolfson, J.; Kandyla, M.; Nelson, K. A., Extended Two-Temperature Model for Ultrafast Thermal Response of Band Gap Materials upon Impulsive Optical Excitation. J Chem. Phys. **2015**, 143, 194705.

(9) Jiang, L.; Tsai, H.-L., Improved Two-Temperature Model and Its Application in Ultrashort Laser Heating of Metal Films. J. Heat Trans. **2005**, 127, 1167-1173.

(10) Baierlein, R.; Gould, H., Thermal Physics. Phys. Today **2000**, 53, 44-45.

(11) Chimier, B.; Utéza, O.; Sanner, N.; Sentis, M.; Itina, T.; Lassonde, P.; Légaré, F.; Vidal, F.; Kieffer, J. C., Damage and Ablation Thresholds of Fused-Silica in Femtosecond Regime. Phys. Rev. B **2011**, 84, 094104.

(12) Stuart, B. C.; Feit, M. D.; Herman, S.; Rubenchik, A. M.; Shore, B. W.; Perry, M. D., Nanosecond-to-Femtosecond Laser-Induced Breakdown in Dielectrics. Phys. Rev. B **1996**, 53, 1749-1761.

(13) Boneberg, J.; Yavas, O.; Mierswa, B.; Leiderer, P., Optical Reflectivity of Si above the Melting Point. Phys. Status Solidi. B **1992**, 174, 295-300.

(14) Velpula, P. K.; Bhuyan, M. K.; Courvoisier, F.; Zhang, H.; Colombier, J. P.; Stoian, R., Spatio-Temporal Dynamics in Nondiffractive Bessel Ultrafast Laser Nanoscale Volume Structuring. Laser Photonics Rev. **2016**, 10, 230-244.

(15) Sun, Q.; Jiang, H.; Liu, Y.; Wu, Z.; Yang, H.; Gong, Q., Measurement of the Collision Time of Dense Electronic Plasma Induced by a Femtosecond Laser in Fused Silica. Opt. Lett. **2005**, 30, 320-322.

(16) Guizard, S.; Semerok, A.; Gaudin, J.; Hashida, M.; Martin, P.; Quéré, F., Femtosecond Laser Ablation of Transparent Dielectrics: Measurement and Modelisation of Crater Profiles. Appl. Surf. Sci. **2002**, 186, 364-368.

(17) Fox M. Optical Properties of Solids. **2002**.

(18) Gunnella, R.; Zgrablic, G.; Giangrisostomi, E.; D'Amico, F.; Principi, E.; Masciovecchio, C.; Di Cicco, A.; Parmigiani, F., Ultrafast Reflectivity Dynamics of Highly Excited Si Surfaces below the Melting Transition. Phys. Rev. B **2016**, 94, 155427.
